# Developmental Profiles of Young Children Referred for Concern for Autism Spectrum Disorder: DBPNet Study

**DOI:** 10.1007/s10803-025-06777-0

**Published:** 2025-03-22

**Authors:** Nancy Roizen, Sandra Friedman, Douglas Vanderbilt, Jaclyn Cacia, Jill Fussell, Robin Hansen, Johannes Hofer, Georgio Sideridis, Ruth E. K. Stein, William Barbaresi

**Affiliations:** 1 Department of Pediatrics, Rainbow Babies and Children’s Hospital, 10524 Euclid Ave Suite 3150, Cleveland, OH 44106 USA; 2 Department of Pediatrics, University of Colorado School of Medicine/Children’s Hospital Colorado, Aurora, CO USA; 3 Department of Pediatrics, Children’s Hospital of Los Angeles, Los Angeles, CA USA; 4 Department of Pediatrics, The Children’s Hospital of Pediatrics, Philadelphia, PA USA; 5 Department of Pediatrics, University of Arkansas for Medical Sciences, Little Rock, AR USA; 6 Department of Pediatrics, University of California, Davis, Sacramento, CA USA; 7 Institute for Neurology of the Senses and Language, Hospital of St, John of God, Linz, Austria; 8 International Precision Child Health Partnership, Boston Children’s Hospital, Boston, Massachusetts USA; 9 Albert Einstein College of Medicine, Department of Pediatrics Bronx, Children’s Hospital at Montefiore, NY, USA; 10 Department of Pediatrics, Boston Children’s Hospital, Boston, MA USA

**Keywords:** Autism, Developmental profiles, Language, Cognition, Adaptive, Young children

## Abstract

The aim of this study was to compare differences in cognitive, adaptive, and language function in young children referred for concerns for autism spectrum disorder (ASD) who are diagnosed with ASD vs those not diagnosed with ASD (no ASD). This prospective diagnostic study of 349 children < 6 years of age, with 250 diagnosed with ASD and 99 with no ASD, was conducted at 8 diagnostic centers. There were no differences in cognition between those diagnosed with ASD and those with no ASD. As compared to those with no ASD, children with ASD had significantly lower language and adaptive functioning. Children with no ASD had language and adaptive functioning similar to their cognitive function. Differences between developmental domains were also compared within the ASD and no ASD groups. There were no differences between any 2 areas of function in the no ASD group. However, there were significant differences within the ASD group, with cognitive function significantly higher than both language function and adaptive function. This study suggests that a discrepancy between adaptive and language skills beyond that expected based on cognitive function should heighten concern for ASD. Beyond the categorical diagnosis of ASD, it is important to assess all these developmental domains to develop comprehensive plans for interventions.

In 1970, 1 to 2 of every 10,000 children were diagnosed with autism (Christensen et al., 2016) compared to 278 of every 10,000 8-year-old children in 2020 (Center for Disease Control, 2023). We currently know that the earlier the identification of autism spectrum disorder (ASD) and initiation of ASD-specific evidence-based interventions the better the outcomes (Dawson et al., 2010). Consequently, screening for ASD has been recommended with a standardized screening tool such as the Modified Checklist for Autism in Toddlers (M-CHAT) at 18 and 24 months of age (Hyman et al., 2020). Children who screen positive for ASD need further evaluations to determine their diagnosis, as a recent meta-analysis of the M-CHAT reported a pooled positive predictive value of 57.7% in the general population (Aishworiyar et al., 2023). However, many children referred with a concern for ASD ultimately will be diagnosed with other developmental challenges that warrant intervention (High et al., 2022). Thus, despite *an initial* concern for ASD, it is essential to understand differences in developmental profiles of those diagnosed with ASD or not diagnosed with ASD (no ASD) to guide efficient assessments and access to appropriate treatments.

There is a paucity in ASD studies of the developmental profiles of young children with ASD. There are no studies comparing comparing the developmental profiles of those with ASD and with no ASD in young children referred for a concern for ASD. Dawson et al., (2010) reported on the Early Start Denver Model of intervention for children with ASD between 18 and 30 months of age using the using the Vineland Adaptive Behavior Scales (VABS-3) and the Mullen Scales of Early Learning (MSEL) comparing those with the intervention with those without the intervention in 44 children. In a retrospective study of 191 children with a mean age of 65.9 months referred for evaluation of possible ASD, analysis of Vineland Adaptive Behavior Scales (VABS-3) and Behavioral Assessment System for Children (BASC-3) scores revealed that those diagnosed with ASD had poorer scores in social and adaptive function on the BASC-3 and all subscales of the VABS-3 (Patel et al., 2024). Some studies which hae included developmental testing of children with ASD have been focused on validating evaluation tools in young children such as the First Year Inventory for 12 month olds (Lee et al., 2021) and LENA (Language Environment Analysis) (Sulek et al., 2022). Most existing studies in children with ASD are focused on school-age children and rarely include multiple developmental domains (Frazier et al., 2014; Liss et al., 2001). Studies of children with ASD who have cognitive function in the average range report deficits in adaptive functioning (Liss et al., 2001; Tillman et al., 2019; Alvarez et al., 2020). In addition, studies report significant discrepancies between cognitive function and language skills (Kjellmer et al., 2018; Norrelgen et al., 2015). These studies suggest that the social communication problems among children with ASD affect both adaptive and language functions.

The literature to date has not provided information comparing developmental profiles of young children referred for possible ASD who ultimately do or do not receive an ASD diagnosis. Therefore, the aim of this prospective diagnostic study is to compare at the time of diagnosis: (1) cognitive, language, and adaptive function between groups of young children diagnosed with ASD and no ASD; and (2) differences across these areas of function within the groups diagnosed with ASD and no ASD.

## Methods

### Study Population and Procedures

Children ages 18 months-5 years, 11 months referred to academic medical center clinics for a concern for possible ASD were consecutively enrolled between May 2019 and February 2020. This study was approved by the Institutional Review Boards of the Children’s Hospital of Philadelphia (Developmental-Behavioral Pediatrics Research Network (DBPNet) coordination center and the 8 participating sites 7 DBPNet and 1 Austrian site each of which provides subspecialty assessment for children with ASD. Participants were excluded if they had a prior multidisciplinary team diagnosis of ASD, or the child was not English speaking (not German speaking at the European site). Clinicians were board eligible or certified in DBP (Developmental-Behavioral Pediatrics) and/or NDD (Neurodevelopmental Disabilities) or physician specialists trained to diagnose and treat ASD at the European site. Using usual clinical protocols specific to each site, a clinical assessment was completed that included a detailed history and demographics (age, sex, race/ethnicity, caregiver education level, and insurance type), physical examination, behavioral observations, an Autism Diagnostice Observation Schedule (ADOS-2) and site-specific selection of other measures. These evaluations included the some of the following measures: (1) *cognitive* (Bayley Scales of Infant Development (Albers & Grieve, 2007), Mullen Scales of Early Learning (Mullen, 1995), Differential Ability Scales (Elliott et al., 2018), Stanford–Binet Intelligence Scales (Roid & Pomplun, 2012), Wechsler Preschool and Primary Scale of Intelligence (Wechsler, 2002), or Wechsler Intelligence Scale for Children (Wechsler, 2003)); (2) *language* (Preschool Language Scales (Zimmerman et al., 2011), Receptive–Expressive Emergent Language Test (Bzoch et al., 2003), Expressive One-Word Picture Vocabulary Test (Michalec & Henninger, 2011), Receptive One-Word Picture Vocabulary Test (Martin & Brownell, 2010), or Clinical Evaluation of Language Fundamentals (Wiig et al., 2013), and (3) *adaptive* (Vineland Adaptive Behavior Scales (Sparrow et al., 2016) or Adaptive Behavior Assessment System (Harrison & Oakland, 2015)) domains. Since this study was embedded in naturalistic clinical assessments, the types and the number of tests administered varied by site. To compare results across disparate scales with each domain, each of these measures were collapsed into categories based on standard score ranges: average to above average (standard scores > 84 to maximum), borderline (standard scores > 69 to 84), mild impairment (standard scores > 54 to 69), moderate impairment (standard scores > 39 to 54), and severe/profound impairment (standard scores ≤ 39 to minimum) based on tool normative data. DBP clinicians made a Diagnostic and Statistical Manual of Mental Disorders (Fifth Edition) (DSM-5) criteria based diagnosis (ASD-yes or ASD-no) based on all the available data listed above and the results of an Autism Diagnostic Observation Schedule, 2nd Edition (ADOS-2) (Lord et al., 2006, 2012). Included in the analysis are all the children who had at least one data point for a developmental domain. Each analysis used fully available data using pairwise deletion in that a subject is not deleted from an analysis if they have a missing value in one variable because they may have a valid value in another variable (in multivariate models).

### Analysis Plan

Descriptive statistics characterize participant demographics. Developmental profile frequencies were compared between groups in the domains of cognitive, language, and adaptive function. Different instruments were used across sites. In order to establish homogeneity of domain functioning across sites and enable meaningful comparisons between ASD and no ASD groups, quantitative rating scores were converted into five categorical ratings ranging from "0" reflecting severe/profound impairment to "4" reflecting average to above average function. The categorical rating scale was adopted in which participants were rated as follows: 0 = severe/profound impairment, 1 = moderate impairment, 2 = mild impairment, 3 = borderline, and 4 = average to above average. The Mplus (https:www.statmodel.com) and Stata (https://www.stata.com) packages were utilized in the present report. The level of significance was set to 5% for a two-tailed test.

Since specific assessments were administered based on clinical practice patterns at each participating site, data on each developmental domain were not available for all participants. To ensure that missing data across assessments did not bias the diagnosis status, we conducted two analyses. First, we conducted a cross tabulation between the group of children that had complete assessments (for all three domains of functioning) and contrasted them with the group that had missing data on any one of the assessments. Next, we contrasted 2X5 tables for diagnosis (2 levels) and level of functioning in cognitive, language and adaptive domains (5 levels) for the two levels of a third variable, defining missingness. In other words, we tested for the homogeneity of findings in the 2X5 tables for children with complete data versus those who had missing values in any one of the assessments.

## Results

### ASD and No ASD Demographics

Among the 349 children enrolled, there were 250 with ASD and 99 no ASD. Overall, these groups showed no differences in race, ethnicity, or gender (Table 1). Participating children were from diverse backgrounds, with 30% of the no ASD and 42% of the ASD groups being none White. Of the entire sample, 19% were of Hispanic ethnicity. A male predominance was seen in both groups as is typical among children with developmental concerns. Those without ASD were more likely to be publicly insured, [63% vs 49%; p = 0.021] and older [42 vs 39 months; p = 0.042].

**Table 1:** Demographic Differences Between Final Diagnosed No ASD and ASD Groups

Demographics	Total N/%	No ASD N = 99	ASD N = 250	Chi-square/t-test	p-value
Age in months (Mean/SD)	349/100%	42.21(14.573)	38.98(12.834)	2.037*	0.042*
Gender (N/%)	349/100%			1.308	0.253
Female		16(16.2%)	54(21.6%)		
Male		83(83.8%)	196(78.4%)		
Race	348/99.7%	98	250	5.403	0.493
White	212/60.9%	68(69.4%)	144(57.6%)		
Black Native	52/14.9% 2/0.6%	11(11.2%) 0(0%)	41(16.4%) 2(0.8%)		
American	22/6.3%	5(5.1%)	17(6.8%)		
Asian	1/0.3%	0(0%)	1(0.4%)		
Hawaiian	33/9.5%	9(9.2%)	24(9.6%)		
More than one race Not reported	26/7.5%	5(5.1%)	21(8.4%)		
Ethnicity (Hispanic)	67/19%	16(16.35)	51(20.8%)	0.898	0.343
Insurance	347/99.4%	98	249	7.773*	0.021*
Medicaid	184/53.0%	**62(63.3%)**	**122(49%)**		
Private	155/44.7%	36(36.7%)	119(47.8%)		
Military	8/2.3%	0(0%)	8(3.2%)		

Bold indicates significant differences in frequency

### ASD vs No ASD Developmental Domain Differences

As shown in Table 2, there are significant differences in language and adaptive skills between the two group (Language chi-square 21.258 p < 0.001; Adaptive chi-square 19.386 p < 0.001). There was no difference in cognitive skill (Cognitive chi-square 6.282 p = 0.1790).

**Table 2:** Differences Between ASD and no ASD Groups in Cognitive, Language, and Adaptive Functioning

Assessments	No ASD	ASD	Chi-square/t-test	p-value
Cognitive (N/%):	57	163	6.286	0.179
Average to Above	32(56.1%)	63(38.7%)		
Average	8(14%)	41(25.2%)		
Borderline	12(21.1%)	39(23.9%)		
Mild Impairment	4(7%)	13(8%)		
Moderate Impairment Severe/Profound Impairment	1(1.8%)	7(4.3%)		
Language (N/%):	45	143	21.258*	< 0.001*
Average to Above	**12(26.7%)**	**13(9.1%)**		
Average	14(31.1%)	26(18.2%)		
Borderline	13(28.9%)	37(25.9%)		
Mild Impairment	**3(6.7%)**	**49(34.3%)**		
Moderate Impairment Severe/Profound Impairment	3(6.7%)	18(12.6%)		
Adaptive (N/%):	53	165	19.386*	0.001*
Average to Above	10(18.9%)	18(10.9%)		
Average	**28(52.8%)**	**47(28.5%)**		
Borderline	**14(26.4%)**	**76(46.1%)**		
Mild Impairment	**0(0%)**	**21(12.7%)**		
Moderate Impairment Severe/Profound Impairment	1(1.9%)	3(1.8%)		

Bold indicates significant differences in frequency

**Fig. 1:**
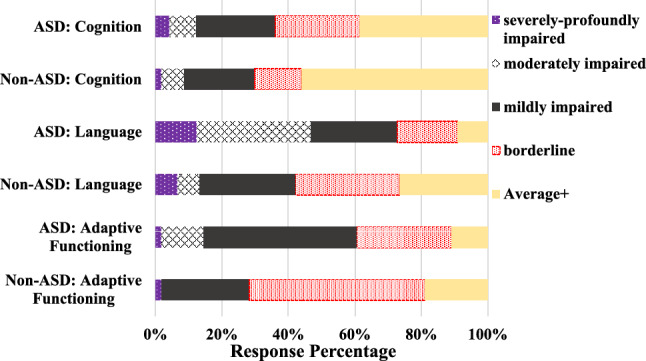
Levels of cognitive, language, and adaptive function between those with ASD and with no ASD

Further investigation of between group differences focused on a series of category curve tests. Developmental profile frequencies differed between groups (Fig. 1). These tests contrasted the frequency of responses between groups using omnibus chi-square tests followed by pairwise z-tests. The latter was corrected using a Bonferroni correction. As shown in Table 2, there were no significant differences between groups in cognition, although in the no ASD group there were higher occurrence of “average and above average” responders compared to the ASD group (No ASD 56.1% vs ASD 38.7%. In language, however, the no ASD group had a significantly higher presence of “average and above average” responders and the ASD group a higher prevalence of “moderate impairment” compared to the no ASD group. Finally, there was a significantly higher prevalence of “moderate impairment” and “mild impairment” in the ASD group in adaptive functioning, with a higher prevalence of “borderline” and “average and above average” functioning in the no ASD group.

### Within ASD and No ASD Developmental Patterns

Differences between developmental domains were compared within the ASD and no ASD groups to assess for patterns. As shown in Table 3, there were no differences between any 2 areas of function in the no ASD group. In the ASD group, cognitive function was significantly above both language function and adaptive function; however, language function was comparatively lower than adaptive function but not significantly.

**Table 3:** Differences within ASD and no ASD groups in cognitive, language, and adaptive functioning

Assessments	Difference Estimate No ASD	Chi-square/t-test	p-value	Difference Estimate ASD	Chi-square/t-test	p-value
Cognitive vs Language:	0.156	0.796	0.432	1.000	10.987***	< 0.001
Language vs Adaptive:	0.212	1.365	0.182	− 0.223	− 2.632*	0.010
Adaptive vs Cognitive:	− 0.172	0.926	0.362	− 0.621	− 5.907***	< 0.001

### Missing Data Analysis

Overall, there were complete developmental domains assessment results for 28.4% of the sample. To ensure that the entire sample represented consistent profiles, missing data analysis was conducted. First, results pointed to no significant differences between children who had complete versus missing assessments with regard to their ASD diagnosis [(Chi-square (1) = 3.482, p > 0.05], thus, the pattern of results remained the same regardless of missingness. Finally, there were 15 statistical tests evaluating the homogeneity of the two samples (full data vs. missing) with two significant effects pointing against the conclusion of homogeneous samples. These two significant effects represented 13.3% of the total number of comparisons, slightly above the alpha level. Given the large number of tests conducted, we adjusted the p-value using the Benjamini–Hochberg correction using a false discovery rate equal to the level of significance a = 5%. Results indicated that the two significant effects were no longer significant after applying the correction for family-wise error. Thus, we conclude that there were no systematic effects of missingness in that samples with full and missing cases were largely equivalent regarding the substantive findings of the study for each of the three developmental function domains.

## Discussion

This prospective diagnostic study of a demographically diverse cohort of young children 18 months to 5 years 11 months of age referred for evaluation of ASD concerns demonstrates lower adaptive and language skills but similar cognitive levels in those diagnosed with ASD compared to those in the no ASD group. Comparison of the 3 developmental domains of cognitive, adaptive, and language function revealed that the no ASD group had no significant differences across these 3 domains. In contrast, the ASD group had significantly lower adaptive and language function compared to their cognitive function. Both sets of comparisons demonstrate the value of an evaluation of cognitive, adaptive, and language function in all children where there are concerns for ASD to develop goals for intervention, regardless of diagnosis.

Early epidemiological reports of ASD indicated that as many as 70% of individuals with ASD had co-occurring intellectual disability (ID) (Myers et al., 2019; Thurm et al., 2019). Most recently, approximately 38% of 8-year-old children with ASD also have ID (CDC, 2023), which is similar to the percentage of the children in this study with cognition below standard scores of 70. In this study, those referred with a question of ASD had similar cognitive function whether diagnosed with ASD or no ASD. However, there were notable differences between ASD and no ASD children in the areas of adaptive and language skills. This gap between higher cognitive function and lower adaptive function in those with ASD was similar to a study by Alvares et al. (2020) who concluded that “high functioning autism” is a misnomer, with cognitive function being a weak predictor of adaptive function. In the Alvarez et al. study (2020), those without cognitive delay had adaptive function significantly below cognitive level while those with cognitive delay had similar adaptive function. In a recent study, higher baseline adaptive skills in young children with ASD increased the odds of being in the group found to have nonpersistent ASD 2–5 years later (Harstad et al., 2023). All the children in the nonpersistent ASD group had an IQ of at least 70 while those in the persistent ASD group had a bimodal distribution of IQ (Harstad, et al, 2023). While there was language impairment in the no ASD group, we found more significant language impairment for those with ASD.

Demographically, this study was consistent with most studies of children with ASD where the populations are predominantly male and white. We found that 49% of children with ASD were insured by Medicaid. In 2019–2021, 36–38%% of U.S children and youth from birth to 18 years were insured by Medicaid (Childstats.gov). Our study was performed at 8 academic medical centers, which tend to accept all patients regardless of type of insurance coverage and thus tend to have relatively higher rates of children insured by Medicaid. Importantly, race and ethnicity of those diagnosed with ASD were diverse and were relatively close to US demographics (Childstats.gov): Hispanic (21% vs 26%), Black (16% vs 14%), Asian (7% vs 6%), and White (58% vs 49%) thereby enhancing the generalizability of our findings.

There were several additional strengths in this study. This prospective study of a large number (n = 349) of young children with concerns for ASDoccurred at seven sites across the US and in one European country. The evaluations took place in naturalistic clinical environments making the results more generalizable to other busy clinical evaluation settings. The demographics of the study participants were representative of the population of the sites and close to the diverse population of the US. The comparison between those diagnosed with ASD and no ASD is unique as most studies compare different domains of functioning only among children with ASD. All study participants had an ADOS administered as part of the diagnostic assessment. Data on function in the three developmental domains were from the date of diagnosis and therefore may not have been impacted by ASD-specific intervention.

This study has several potential limitations. This sample of patients referred to 7 DBPNet sites and 1 international site were not evaluated using a uniform developmental testing protocol but followed the usual clinical evaluation approach of each center. Therefore, not all the children in the study had developmental evaluations of cognition, adaptive, and language function and the tests used were not the same across sites. However, we employed 2 analytic methods to show that missing data were not different between the ASD and no ASD groups. Of note, we adopted a categorical rating scale to be able to compare scores of the different diagnostic tests administered at sites. It is important to note that variations in assessments across sites reflects the diversity of clinical practice in the field.

## Conclusion

In this study of developmental profiles of young children referred for ASD evaluation in the naturalistic environment of a clinical setting, there was no significant difference in cognitive function among young children referred for concern of ASD, whether they ultimately received the diagnosis of ASD or no ASD. However, for those children diagnosed with ASD, adaptive and language function were both lower as compared to those with no ASD while there were no differences in cognitive function. For the children with concern for ASD who ultimately were not diagnosed with ASD, their levels of cognitive, language, and adaptive function were similar. Discrepancy between adaptive and language skills compared to cognitive function should heighten concern for ASD. We contend that above and beyond establishing a categorical diagnosis of ASD, it is important to assess all these developmental domains to obtain a full understanding of child’s strengths and challenges and thus develop comprehensive plans for support, interventions, and monitoring.
